# Low frequency 3D ultra-wide vibration attenuation via elastic metamaterial

**DOI:** 10.1038/s41598-019-44507-6

**Published:** 2019-05-29

**Authors:** Luca D’Alessandro, Raffaele Ardito, Francesco Braghin, Alberto Corigliano

**Affiliations:** 1 Politecnico di Milano, Civil and Environmental Engineering, Milano, Italy; 2 Politecnico di Milano, Mechanical Engineering, Milano, Italy

**Keywords:** Mechanical engineering, Civil engineering

## Abstract

The design of innovative metamaterials with robust and reliable performances is attracting increasing interest in the scientific community because of their unique properties and for their unexplored potential. In particular, dynamical properties of periodic structures are widely studied specifically for their bandgap opening characteristic, which enables the design of structures with unprecedented dynamical behaviour. In the present work an ultra-wide three-dimensional bandgap is presented, with extremely low frequency range of operation. Numerical simulations and analytical models are proposed to prove the claimed properties, together with experiments carried out on a prototype built by means of additive manufacturing.

## Introduction

Periodic structures and metamaterials are gaining increasing interest due to the unprecedented properties and performances that they are able to express in wave propagation control and manipulation. Starting from relevant applications in the electro-magnetic domain^1,2^, in recent years important attention has been devoted to their applications in the vibro-acoustic field^3,4^. Elastic periodic structures, in particular, find numerous applications in wave propagation control ranging over the whole frequency spectrum of the vibration phenomenon^5^: from extremely high frequencies, i.e. THz region for heat transmission^6^, to several applications in the Micro-Electro Mechanical industry^4,7^, to few Hz in the *seismic metamaterials* domain^8–13^. The most important feature of the proposed structures is the bandgap (i.e. the frequency range of prevented wave transmission): wide and complete bandgap means robust control over the wave propagation^4,14–17^. In many cases, complete bandgap are presented in 1D or 2D periodic structures^18–24^, while few cases of complete bandgap in 3D periodic arrangement are described in literature. Those 3D designs are often made of two or more materials^25,26^, to maximize the impedance mismatch and therefore create robust and wide bandgaps^4^, with natural complexities in the prototyping and assembly phase of the material itself. Significant results have also been achieved for 3D periodic structures made of a single material^15,17,27–31^, among which the most performing one in terms of gap to mid-gap ratio presented by the authors^16^. One of the major drawbacks of the described periodic structures, particularly for macro-scale problems (i.e. groundborne vibrations, earthquake), is the dimension of the unit cells, which are often too big for the practical realization of the proposed solutions in real industrial cases^4^. This work addresses the objective of achieving unprecedented performances over mechanical wave control, with a single material, for all possible directions of incidence in the three-dimensional space, keeping the unit cell dimension of the periodic structure at a sub-wavelength regime. The principle adopted relies on the *separation of modes*^16^: a specific distribution of structural elements, arranged either as *masses* or *elastic ligaments*, is designed, to endow the overall structure with an ultra-wide bandgap located at low frequency. Numerical calculations and analytical modelling are proposed to describe the structure, as well as experimental tests conducted on a prototype manufactured via additive manufacturing.

## Results

**Figure 1:**
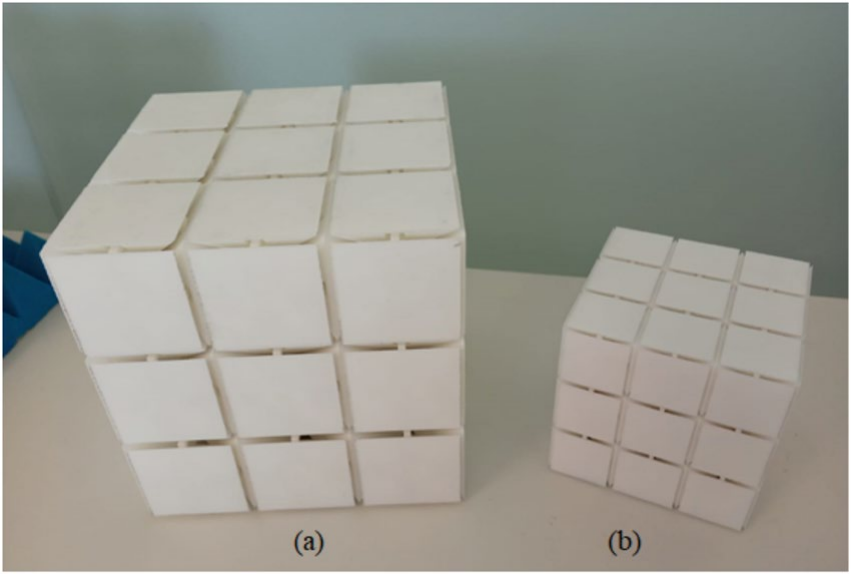
Prototypes of the mechanical metamaterial presented: they are composed of 3 × 3 × 3 unit cells repetition. In prototype (**a**), the unit cell side is 5 cm; in (**b**) it is 3 cm. The prototypes have been manufactured in Nylon PA12^36^ via Selective Laser Sintering technique.

The results are referred to a couple of periodic structures, characterized by the same shape but with different size: the “large” structure, see Fig. 1a, is endowed with a 50 mm unit cell, whereas the “small” structure, see Fig. 1b, has a unit cell with sidelength equal to 30 mm. In both cases, a 3 × 3 × 3 unit cell configuration is considered.

**Figure 2:**
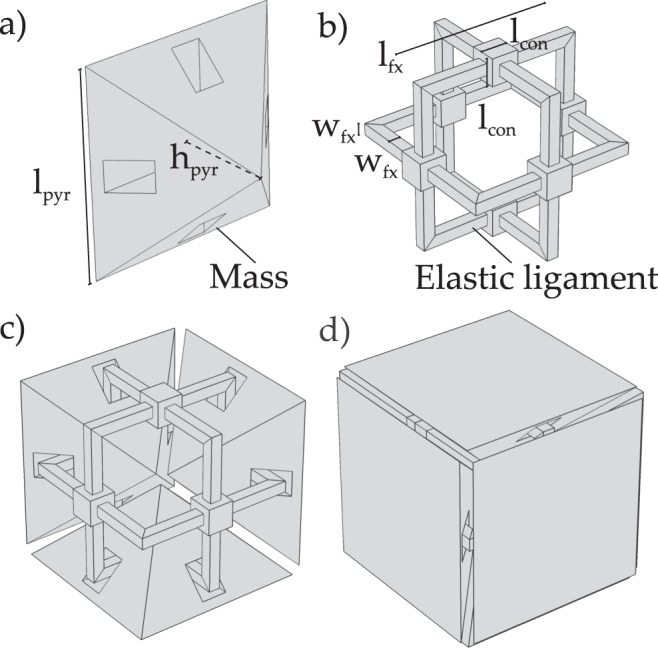
Unit cell description of the periodic structures of Fig. 1: (**a**) elements composing the unit cell which play the role of *masses*; (**b**) elements composing the unit cell which play the role of *elastic ligaments*; (**c**) partial composition of the unit cell; (**d**) total composition of the unit cell.

As described in the introduction, the periodic structures are composed of two types of elements, each one with specific mechanical role: *masses* (refer to Fig. 2a) and *elastic ligaments* (refer to Fig. 2b). The two are organised to form a simple cubic unit cell, as shown in Fig. 2c,d.

The proportions among unit cell components are described in reference to the parameters introduced in Fig. 2, and they are shown in Table 1, where *a* is the overall size of the unit cell. The proportions are not the same for the two prototypes, since it is necessary to comply with some specific constraints connected to the manufacturing method: as an example, the minimum width of the ligaments cannot be smaller than 2 mm, in order to avoid large inhomogeneities of the dimension and, possibly, unexpected failures at the end of the production process.

**Table 1:** Geometric dimensions of unit cell components described in Fig. 2a,b. The parameter *a* is the unit cell characteristic dimension, which represents the overall size of it.

Prototype	Quantity	*a*	*l*_*pyr*_	*h*_*pyr*_	*w*_*fx*_	*l*_*fx*_	*l*_*con*_
Large (a)	Dimension with respect to *a*	1.00*a*	0.92*a*	0.46*a*	0.06*a*	0.85*a*	0.14*a*
	Dimension [m]	0.0500 m	0.0460 m	0.0230 m	0.0030 m	0.0425 m	0.0070 m
Small (a)	Dimension with respect to *a*	1.00*a*	0.92*a*	0.46*a*	0.0667*a*	0.80*a*	0.1667*a*
	Dimension [m]	0.0300 m	0.0276 m	0.0138 m	0.0020 m	0.0240 m	0.0050 m

### Unit cell analysis for the large prototype

**Figure 3:**
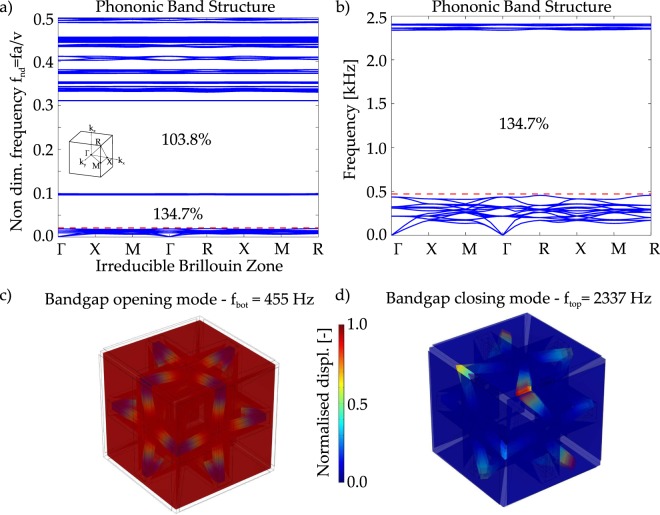
Phononic band structure of the periodic structure of Fig. 2. (**a**) Non dimensional diagram: *f*_*nd*_ is the non dimensional frequency, *f* is the dimensional frequency and $v=\sqrt{E/\rho }$ is the sound velocity in the medium. (**b**) The dimensional graph with focus on the first bandgap, calculated for the topology shown in Fig. 1: *a* = 5 cm and Nylon PA12^36^ as material (*E* = 1586 MPa, Poisson’s ratio 0.4, *ρ* = 1000 kg/m^3^, *v* = 1259 m/s); the horizontal red dashed line is the analytical approximation of the bandgap opening frequency obtained with 1D spring-mass chain model. The considered IBZ is provided. (**c**) First bandgap opening mode; (**d**) first bandgap closing mode.

The large prototype, Fig. 1a, has the same overall dimension of the previously considered cases^15,16^, with a side length of the unit cell equal to 5 cm. The dispersion diagram referred to the topology of Fig. 2 is calculated by means of the Solid Mechanics Module of COMSOL Multiphysics v5.3, and it is shown in Fig. 3a in non dimensional units. The first bandgap limits, in non dimensional units (refer to caption of Fig. 3a for details), are: *f*_*nd*,*op*_ = 0.018088 and *f*_*nd*,*cl*_ = 0.092801, characterising an ultra-wide frequency range of operation of gap to mid-gap ratio equal to 2(*f*_*cl*_ − *f*_*op*_)/(*f*_*cl*_ + *f*_*op*_) = 134.7%. The most interesting fact is that the frequency limits are below the unit, meaning that this bandgap is located at sub-wavelength regime. This property is fundamental for practical applications since it permits to control mechanical wave propagation with a periodic structure whose unit cell characteristic dimension is smaller than the incident wavelength. Being the opening frequency *f*_*nd*,*op*_ = 0.018088 means that the bandgap starts to operate as a frequency filter facing a travelling wave that has a wavelength of *a*/0.018088 = 55*a*, therefore 55 times bigger than the size of the unit cell.

The opening and closing modes of this first bandgap are shown in Fig. 3c,d: the opening mode is characterized by the *mass* elements that coincide with the modal masses, while the *elastic ligaments* play the role of modal stiffnesses, experiencing a flexural-type of deformation; the closing mode, instead, is characterized only by the out of plane vibration of the elastic ligaments, with the pyramidal masses that do not participate in the mode. This is due to the arrangement and the design of the elements in the unit cell, which exploits the *separation of modes*^16^, guaranteeing a wide, complete and robust three-dimensional bandgap.

It is interesting to give an analytical demonstration to the low frequency range of operation of this bandgap. A simple mono-atomic spring-mass chain^4^ model is introduced to describe the opening frequency (refer to Fig. 3c): the stiffness of the *elastic ligament*, *k*_*mono*_, can be computed considering the single frame stiffness; while the mass parameter *m*_*mono*_ is calculated considering the total mass of the double pyramids as a point mass. The stiffness of the single frame is computed on the basis of axial, bending and shear compliance^16^: ${k}_{mono}=\mathrm{2/}({l}_{fx,el}/EA+{l}_{fx,el}^{3}\mathrm{/12}EI+{l}_{fx,el}/G{A}^{\ast })$, where $I=\mathrm{1/12}{w}_{fx}^{4}$, $A={w}_{fx}^{2}$, A* = *A*/1.2, *l*_*fx*,*el*_ = (*l*_*fx*_ − *w*_*fx*_ − *l*_*con*_)/2. The stiffness is *k*_*mono*_ = 52.1 N/mm. The mass of the double pyramid is *m*_*mono*_ = 26.9 g. The first bandgap opening frequency is calculated via mono-atomic spring-mass chain^32^: ${f}_{an}=\mathrm{2/(2}\pi )\sqrt{{k}_{mono}/{m}_{mono}}=443.4$ Hz and *f*_*nd*,*an*_ = *f*_*an*_*a*/*v* = 0.017611. This limit is represented by red dashed horizontal line in Fig. 3a,b. The difference between the numerical and analytical calculation for the bottom frequency limit is of 2.6%.

### Finite structure analysis for the large prototype

**Figure 4:**
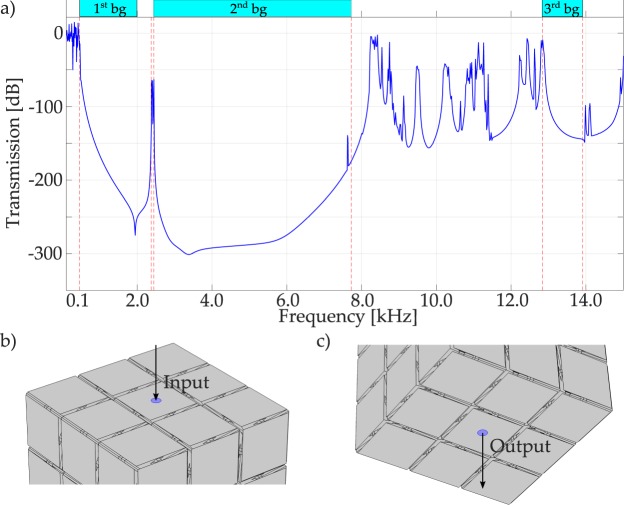
Numerical transmission spectrum for the large 3D structure of Fig. 1a considering a linear elastic behaviour for the Nylon.

The prototype of Fig. 1a, 3 × 3 × 3 unit cell configuration, is numerically studied and experimentally tested to verify the wave filtering properties. The transmission spectrum is numerically and experimentally evaluated by considering the input force in the central portion of the top area of the prototype of Fig. 1a (refer to Fig. 4b), and the output area in the bottom one (refer to Fig. 4a), and measuring in both areas the accelerations, calculating the transmission in dB scale.

First, the numerical linear elastic transmission spectrum referred to the structure of Fig. 1a is calculated along the Irreducible Brillouin Zone path Γ − *X*^33^, and is shown in Fig. 4a: this spectrum confirms the filtering behaviour of the bandgap regions introduced before, with attenuation values that reach 15 orders of magnitude (i.e. 300 dB). The transmission spectrum confirms the local nature of the modes between the first and the second bandgap, as shown in Fig. 3d: even in the case of linear elastic behavior (i.e. in the absence of damping), the second passband is practically canceled, because the transmission spectrum shows a 60 dB minimum attenuation in that frequency range. This is due to the fact that the modes in that passband are not effectively activated in view of their local nature. Such a result is interesting for practical application, since the subsequent bandgaps are merged and the filtering properties of the periodic structure are enhanced^16^, regardless of the material damping.

**Figure 5:**
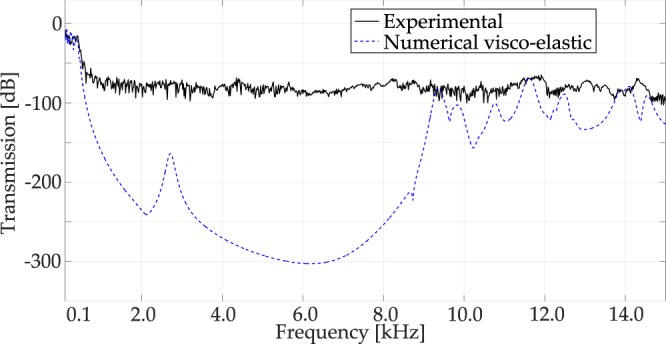
Transmission spectra for the large 3D structure of Fig. 1a, in the Γ − *X* direction^33^: in black solid line the experimental result, in blue dashed line the numerical visco-elastic one.

To confirm the filtering properties of the periodic structure, an experimental test is conducted to measure the transmission between input and output areas as defined in Fig. 4b,c. The prototype shown in Fig. 1a has been tested using an inertial actuator, as better specified in the Methods section. Tests are carried out using 60 s long actuation signal, characterised by a white noise spectrum in the range 0.1–15.0 kHz. The measured transmission spectrum is shown in Fig. 5 in black solid line: this curve displays almost complete transmission for the periodic structure in the first passband, and subsequently a drop to 75 dB of attenuation through the entire spectrum. It is worth noticing that the maximum attenuation that can be measured with the current experimental setup is 75 dB (i.e. 3.75 orders of magnitude of difference between output and input signals). As expected, the second passband is overpassed and the two bandgaps are merged. Moreover, the experimental transmission spectrum is flat even after the end of the second bandgap, showing the typical behavior of a low-pass mechanical filter^16^. Such a response can be explained taking into account the visco-elastic behaviour of Nylon PA12, that plays a fundamental role in the high frequency regime. To investigate that fact, the transmission plot is evaluated numerically on the basis of a model which includes a Standard Linear Solid viscoelastic behavior^34,35^. Such a constitutive law has been thoroughly characterized in previous works^17^ and the coefficients of the Maxwell’s branch have been identified as follows: relaxation time *τ*_*Maxwell*_ = 1.5306*e*–4, Young’s modulus E_*Maxwell*_ = 490 MPa. The numerical outcomes are shown in Fig. 5 blue dashed line. In order to compare the numerical and experimental data, it is necessary to remind the above mentioned limitation: the part of the experimental data beyond the bandgap opening is just indicating that the attenuation is larger than 75 dB, but the real value cannot be measured in view of the experimental resolution. The numerical results are in good agreement with the experimental outcomes as far as the attenuation does not exceed the above mentioned threshold: this means that the analyses are able to predict correctly the bandgap opening. In the high frequency region, the introduction of dissipative behaviour smooths the peaks, that do not exceed −75 dB, apart from a limited region around 11500 Hz, in good agreement with the experimental results.

**Figure 6:**
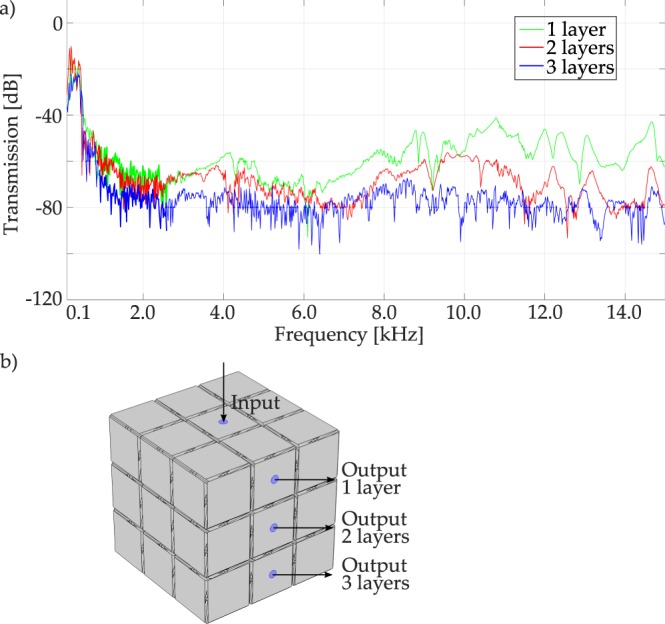
Experimental transmission spectra for the large 3D structure of Fig. 1a, along an inclined direction in the *k*_*x*_ − *k*_*y*_ plane, close to the Γ − *M* path^33^: (**a**) transmission spectra respectively in green, red and blue line for the 1^*st*^, 2^*nd*^, 3^*rd*^ layer; (**b**) the input and output layer areas definition.

To confirm the first experimental results, other directions of wave propagation are considered in the experimental test (refer to Fig. 6b for details about the analysed directions), to roughly represent the IBZ path Γ − *M*, being inclined in one plane parallel to one of the faces of the cubic prototype. The spectra shown in Fig. 6a show that for the first layer (green line) the transmission drops in the low frequency range, and then reaches back measurable values around 40 dB of attenuation at high frequency. The attenuation increases with increasing layer, is never less than 40 dB, reaching the experimental setup limit in the third layer, as already observed in Fig. 5 black solid line. This result is consistent with the fact that the longer the wave propagation path, the larger the attenuation.

### Numerical and experimental results for the small prototype

The small prototype, Fig. 1b, is now considered in order to verify the capability of the proposed metamaterial to filter low-frequency waves even if the size of the unit cell is reduced. The side length of the unit cell is now equal to 3 cm, so that the whole specimen is a cube with 9 cm long sides.

The unit cell analyses are not reported in detail for the sake of conciseness. The opening and closing modes are pretty similar to those shown in Fig. 3c,d, with frequencies equal to *f*_*op*_ = 1251 Hz and *f*_*cl*_ = 5770 Hz: the gap to mid-gap ratio is 129%, aligned with the value for the large prototype. The non dimensional frequencies that define the bandgap region are *f*_*nd*,*op*_ = 0.029809 and *f*_*nd*,*cl*_ = 0.13749. It is important to notice that such values are slightly larger than the ones for the large prototype. This is due to the fact that, in view of production constraints, it is not possible to scale down all the dimensions in the same way. Most importantly, the proportion of the ligament thickness is larger than before, so an increase of the non dimensional frequency is well expected.

**Figure 7:**
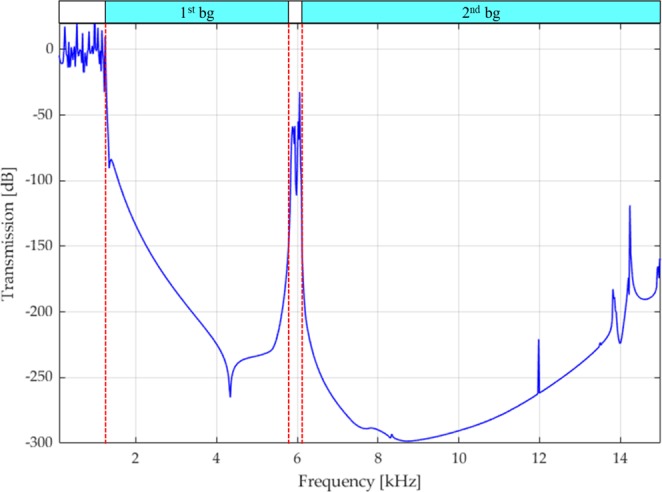
Numerical transmission spectrum for the small structure of Fig. 1b, considering a linear elastic behaviour for the Nylon.

**Figure 8:**
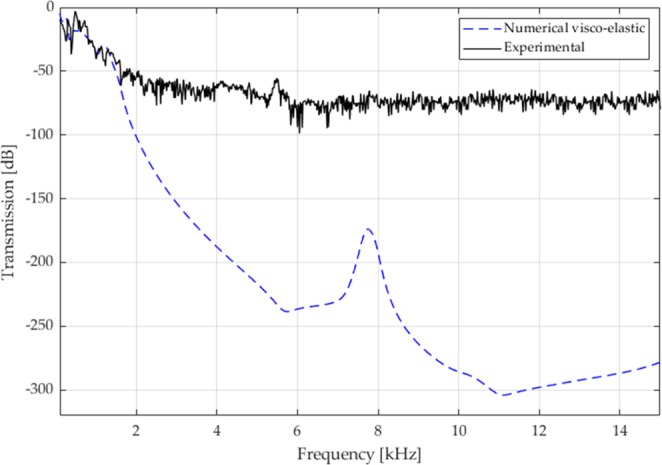
Experimental transmission spectra (black solid line) for the small structure of Fig. 1b, compared to the numerical predictions on the basis of a visco-elastic constitutive model (blue dashed line).

The linear elastic transmission plot, shown in Fig. 7, confirms the width of the first bandgap and the fact that the two subsequent bandgaps are fused: indeed, the transmission plot in the narrow passband is around −50 dB. Consequently, it is reasonable to expect a low-pass mechanical filter also for the small prototype. Such a behavior is evident in the experimental results, shown in Fig. 8. The experiments are carried out with the same setup as for the large prototype, i.e. the transmission between the top and the bottom surfaces is measured by means of accelerometers (refer to Fig. 4b,c). The experimental transmission spectrum is lower than zero also in the low-frequency regime, under 1 kHz, possibly because of damping. After the theoretical bandgap opening, the transmission plot drops to −75 dB, that is the minimum threshold, and stays constant to that value over the remaining frequency spectrum.

As for the large prototype, the simple visco-elastic model is adopted to obtain the numerical prediction of the transmission plot. As shown in Fig. 8, the numerical results match well the experimental outcomes in the low frequency range: the damping effect is correctly simulated. In the frequency range beyond the opening frequency, the spectrum is always by far smaller than −75 dB. A sort of peak is visible around 7800 Hz: that corresponds to the passband, shifted towards the high frequency end because of the increase of apparent elastic modulus for high frequency of excitation. Nonetheless, such a peak gives a 175 dB attenuation, that cannot be detected in the experiments. Therefore, the filtering properties are confirmed both experimentally and numerically.

## Discussion

In this work a three-dimensional elastic metamaterial is described, endowed with a complete, robust and 3D bandgap located at low frequency, in the sub-wavelength regime. This design is supported via a simple analytical model and a design strategy which relies on *separation of modes*, as well as numerical dispersion calculations referred to the unit cell. The wave attenuation is quite large and it exceeds the threshold connected to the experimental resolution. As explained in a previous paper^16^, on the basis of specific experimental tests, the typical attenuation due to material damping does not exceed 40 dB across a 15 cm long specimen. This confirms that the huge attenuations obtained for the proposed metamaterial are caused by the specific geometric features, rather than the material properties.

Experimental tests are conducted along different directions of wave propagation, proving that the 3D filtering property is robust and also more performing with respect to what is predicted from the unit cell analysis, thanks to the global and local arrangement of modes in the periodic structure.

With respect to previous realizations^15,16^, the proposed metamaterial is still endowed with an ultra-wide 3D complete bandgap, with the important advantage of a substantial shift towards the low frequency regime. Indeed, both numerical analyses and experimental tests show that the filtered waves are characterized by a maximum wavelength that is 55 times larger than the size of the unit cell for the large prototype and 33 times for the small prototype. The latter is built to verify the property of low-frequency bandgap even if the overall dimension is small. Indeed, the bandgap opens at 1251 Hz for an overall dimension of 9 cm; conversely, the layout presented in the previous work^16^ enabled the realization of bandgap starting from 1652 Hz with an overall dimension of 15 cm. Therefore, the peculiar property of the proposed metamaterial is confirmed. It is worth mentioning that the sub-wavelength regime can be achieved with different metamaterials, such as locally resonant metamaterials (LRM^4^), that allow the user to select the bandgap opening frequency so that the wavelength is by far larger than the unit cell size. On the other hand, the proposed metamaterial shows some interesting features that are not feasible with LRM, since it enables the creation of huge bandgaps in the sub-wavelength regime and it approaches the behavior of a low-pass mechanical filter. As a consequence, in the presence of a specific target in terms of frequency to be filtered, the unit cell of the proposed metamaterial can be by far smaller than that of previous proposals. Such a feature enables several practical applications in the industrial and infrastructure sectors and paves the way for future research and technical developments in the field of metamaterials applied to vibration control.

## Methods

The prototype is fabricated by means of the Selective Laser Sintering technique^36^, which permits the realization of any 3D geometry. More specifically this technique permits the realization of suspended structures through the plane sintering process thanks to the bearing capacity of the Nylon powder with respect to the sintered one.

To acquire the transmission spectra, the prototype is placed on a bubble wrap that isolates it from the environmental vibrations. A VibeTribe-Mamba with 20 W power and a frequency range from 40 Hz to 22 kHz is used as actuator, while two PCB Piezotronics 353B15 accelerometers, with sensitivity of 10 mV/g and resonant frequency of 70 kHz, are glued in the input and output surfaces respectively and are used as sensors. The data acquisition chain is completed with an 8-channel PCB 483C05 ICP® Sensor Signal Conditioner, and a NI 9205 module, with 16-bit resolution.
